# Attention U-Net-based semantic segmentation for welding line detection

**DOI:** 10.1038/s41598-025-00257-2

**Published:** 2025-05-01

**Authors:** Hunor István Lukács, Bence Zsolt Beregi, Balázs Porteleki, Tamás Fischl, János Botzheim

**Affiliations:** 1 Department of Artificial Intelligence, Faculty of Informatics, ELTE Eötvös Loránd University, Pázmány P. Sétány 1/A, Budapest, 1117 Hungary; 2 Robert Bosch Kft, Gyömrői út 104, Budapest, 1103 Hungary

**Keywords:** Weld detection, Attention U-Net, Semantic segmentation, Industrial AI, AI-based automatization, Machine vision, Computer science, Information technology

## Abstract

In industrial processes, quality assurance through methods such as visual inspection is essential for ensuring process stability. Traditional manual visual inspection is a time-consuming and costly endeavor. If the opportunity arises, replacing manual visual inspection with AI could lead to significant efficiency gains. However, simply judging the correctness or incorrectness of a process is often insufficient; quantitative attributes must also be associated with visual inspection. This paper proposes a solution for replacing manual visual inspection with AI specifically for welded joints. The aim is not only to detect the presence of weld joints but also to assess their geometric dimensions. Leveraging a proposed Attention U-Net architecture in combination with rule-based metrics, the proposed method offers a novel solution for identifying welding lines in images. By integrating semantic segmentation techniques, the method effectively distinguishes weld joint elements, while rule-based metrics facilitate the identification of critical cases requiring human intervention. Experimental results demonstrate the method’s capability to automate a significant portion of inspection tasks, thereby reducing the reliance on manual labor and enhancing overall process efficiency and reliability.

## Introduction

In industrial processes, ensuring quality through techniques like visual inspection is crucial for maintaining process stability. Ensuring product quality and safety in modern manufacturing heavily relies on accurate welding line identification. However, traditional manual inspection is labor-intensive, time-consuming, and error-prone, especially with the increasing volume of image data from automated welding. Replacing this manual process with AI-supported automation could enhance efficiency and eliminate a costly and time-consuming workflow. However, in processes such as welding, it is often insufficient to merely determine whether a weld has been created between two parts; it is also necessary to assess whether the correct amount of material has been welded and whether the weld has been positioned accurately in the target area. Additionally, there should be no foreign material around the weld, such as excess welds or “spatter,” as metallic debris can cause short circuits in the operation of electronic sub-assemblies. Such anomalies are the focus of anomaly detection.

Laser beam welding^1,2^ (LBW) is widely used for strong and precise welds, but process variations can lead to inconsistencies. Therefore, automated weld line inspection is crucial for maintaining LBW quality.

The paper^3^ highlights how Selective Laser Melting (SLM) in additive manufacturing can introduce defects, stressing the importance of reliability. It proposes a new method using a lightweight U-Net-based neural network to capture melt pool signatures, improving real-time control and demonstrating better results than traditional image segmentation in terms of speed and accuracy.

The paper^4^ provides a comprehensive review of deep learning-based welding image recognition (DLBWIR). It surveys state-of-the-art research, focusing on key technologies, applications, tasks, and available public datasets.

Identifying and filtering risky products through outlier detection is crucial for ensuring quality and reducing waste in manufacturing, as demonstrated in surface defect detection studies^5^ and automotive applications^6,7^.

This paper focuses on identifying and filtering out risky LBW products, particularly those with improper welds in industrial applications. The resolution of this challenge offers significant real-world industrial benefits, as it involves ensuring product safety and performance in critical automotive components. The primary contribution of this work is an AI-based method for automating weld line detection, which reduces reliance on manual inspection by over 90% and improves the speed and accuracy of quality assurance, leading to faster identification of defective parts and enhanced manufacturing efficiency.

## Problem statement

Welding is important in industrial joining technology as it establishes both mechanical and electrical connections between two metallic materials, enabling the creation of strong, durable structures that are essential for various applications, including automotive manufacturing, where reliable electrical connections are crucial for the functionality of components. However, manually identifying welds and ensuring their correctness is a time-consuming task, especially given the large volume of data generated in these environments.

In modern manufacturing settings, the images of welds used for quality assurance and inspection are sourced from a variety of machines and production lines. Each machine may utilize different components and products, leading to inherent variability in the captured images. This variability is further complicated by the use of distinct camera systems across different production lines, which can introduce deviations in image quality, perspective, and lighting conditions. Consequently, the dataset used for training and validating automated inspection systems must account for these discrepancies to ensure robust performance. The diversity in equipment and components can result in significant differences in the appearance of welds, making it essential to employ appropriate data preprocessing and augmentation techniques. Often, even under identical welding and camera system settings, the smoke generated by the technology affects image quality and simultaneously makes it difficult for the human eye to recognize the correctness of the weld joint.

Filtering out risky products is crucial, as failing to do so may lead to malfunctions. Currently, the state-of-the-art method in the industry relies heavily on human resources, where decisions regarding laser welding quality are made manually based on images. This article introduces an AI-based method designed to reduce the need for manual inspection of products.

The proposed automatic method combines the U-Net architecture with rule-based techniques to identify cases that require expert intervention. This approach aims to replace manual processes in over 90% of cases. The proposed solution is based on image analysis, where the U-Net architecture performs semantic segmentation of the images to predict the weld joint elements. This prediction is then associated with rule-based metrics, enabling the assessment of weld joint quality.

## Dataset

**Fig. 1:**
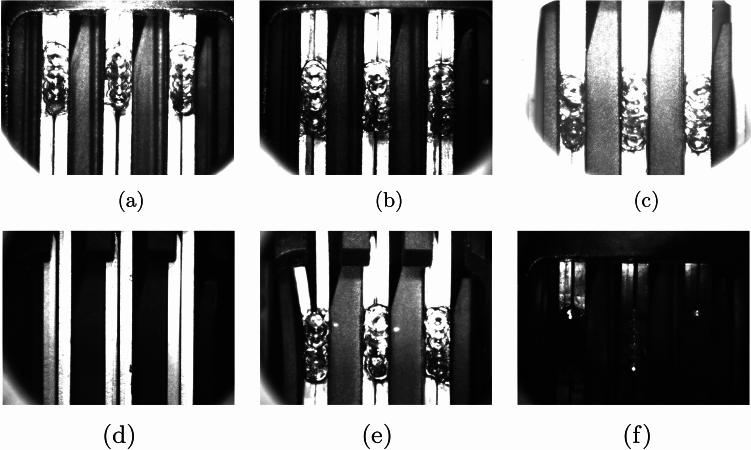
Weld positions on pins (top, mid, bottom) and not accepted weldings (missing weld, bent pin leg, dark/smoky image).

Our dataset comprises images of LBW welding used in automotive components, sourced from various production lines and captured with different camera systems. Figure 1 provides representative examples from the dataset, showcasing the variability in welding quality and the challenges encountered during analysis. All images in the dataset are grayscale (black and white), which simplifies processing while retaining sufficient detail for analysis. Each image contains three pins, consistently positioned across all samples. Each pin is expected to have one weld, with gaps present between the pins and the remaining areas of the image representing the background.

The first row of Fig. 1 illustrates correctly welded cases, with weld positions varying across the top, middle, and bottom of the pins. This positional variability introduces distributional differences in the dataset, which were addressed through data augmentation techniques such as flipping to balance the dataset and reduce potential positional biases during training.

The second row exhibits different cases that do not meet the requirements and need to be filtered out. The left image portrays a situation where the weld is absent from all three pins. In the middle image, the left pin leg appears bent, indicating a need for filtration. The right image depicts a case of excessive darkness, possibly due to welding smoke, necessitating further examination by domain experts due to the uncertainty involved.

**Fig. 2:**
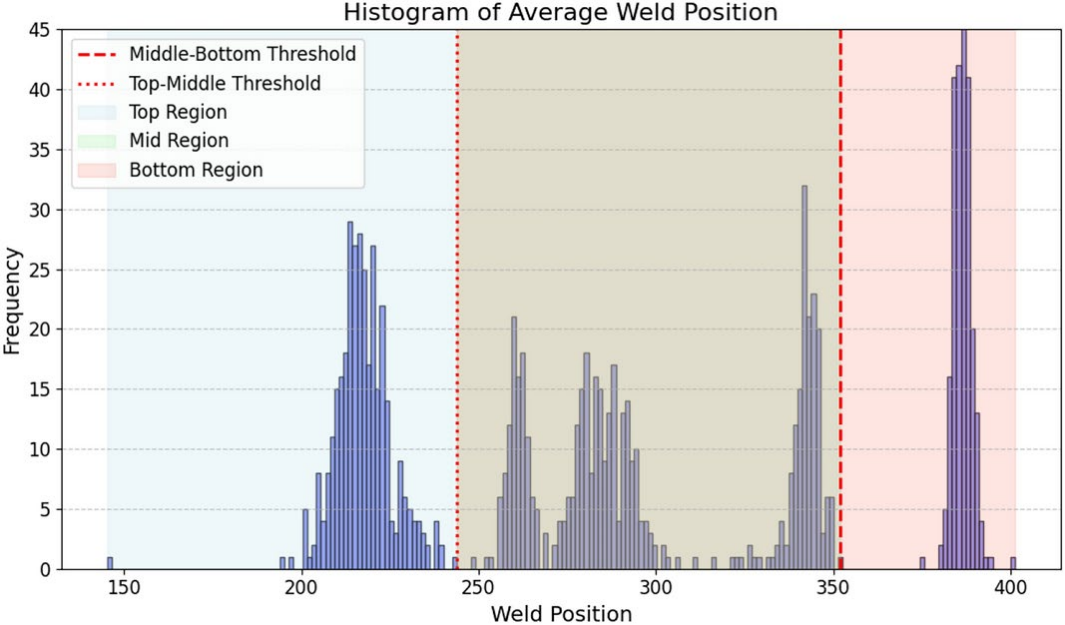
Histogram of weld positions.

The labeled dataset comprises 1,452 original images paired with their corresponding semantic masks. Figure 2 illustrates the histogram of images from our dataset organized by weld position. The horizontal axis represents the midpoint of the weld positions, indicated as pixel values, with the top of the image designated as 0 pixels. A lower average midpoint implies that the weld is located higher in the image.

The distribution of classes based on the weld position is as follows: 32% for the top class, 46% for the mid class, and 22% for the bottom class. To achieve a better balance between the top and bottom classes and to increase the dataset size, a data augmentation technique involving flipping was utilized. Furthermore, the dataset contains 60 images where the weld is missing (e.g., Fig. 1d).

**Fig. 3:**
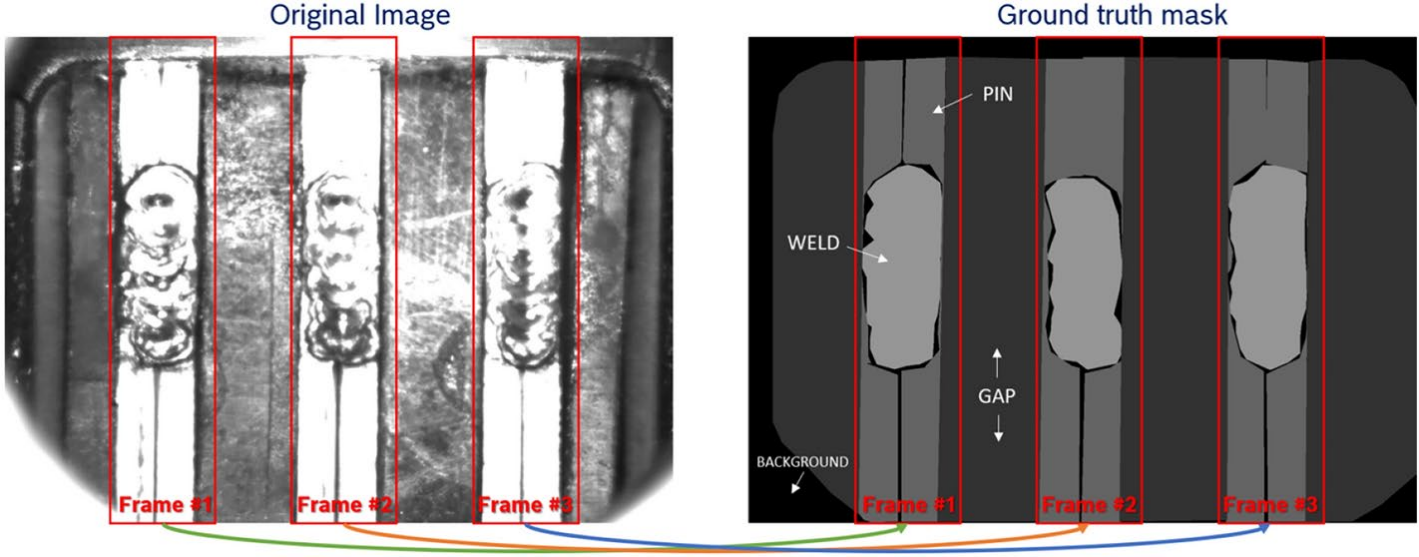
Pin separation.

Similarly to the weld position, the dataset is also distinguished based on the average image brightness, and separated in to 3 different classes: dark (19%), moderate (12%) and bright (69%). The image depicted in Fig. 1f is classified as dark, while Fig. 1a, b, d, e fall into the moderate category. In contrast, Fig. 1c is categorized as bright.

The data across the various classes (no weld, weld top, weld mid, weld bottom, dark, moderate and bright) is evenly distributed among the training (70%), validation (15%), and test (15%) datasets.

Since training a U-Net model is computationally demanding, the original HD images were down scaled to a size of 800 $\times$ 600. In order to achieve a more precise solution in semantic segmentation, only the most relevant information within the frames is retained, focusing on the three pins. The three frames are positioned at fixed locations on the original images. The training, validation, and test datasets (70%–15%–15%) used for the semantic segmentation task consist of smaller input images of size 130 $\times$ 600 and the corresponding target mask, indicated by the three red frames depicted in the Fig. 3. This semantic segmentation task involves four classes: weld, pin, gap, and background. The labeled dataset comprises, 1452 original images paired with their corresponding semantic masks. Therefore, after extracting the 3 frames, the dataset consists of 4356 input images and their respective target masks.

## Proposed method

**Fig. 4:**
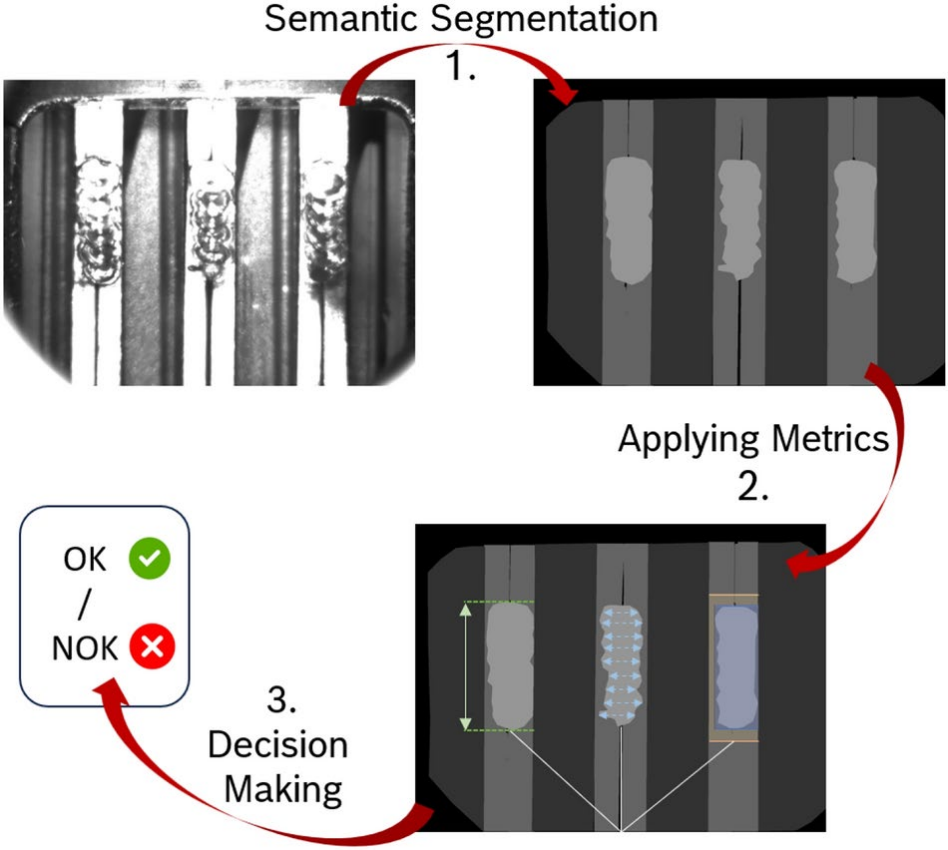
Method overview.

Figure 4 presents a high-level overview of the proposed method, which comprises three main steps: semantic segmentation, metric application, and decision-making.

During the initial step, a U-Net model is employed to generate a semantic mask of the input image. Following this, in the second step, various metrics are applied to assess factors such as weld length, width, brightness, contrast, and pin integrity. Finally, in the third step, the decision-making process occurs. Here, the product associated with the input image is accepted if it meets the criteria defined by each metric. Conversely, if it fails to meet these criteria, it is rejected and referred back to domain experts for further investigation.

## Proposed attention U-Net model for semantic segmentation

**Fig. 5:**
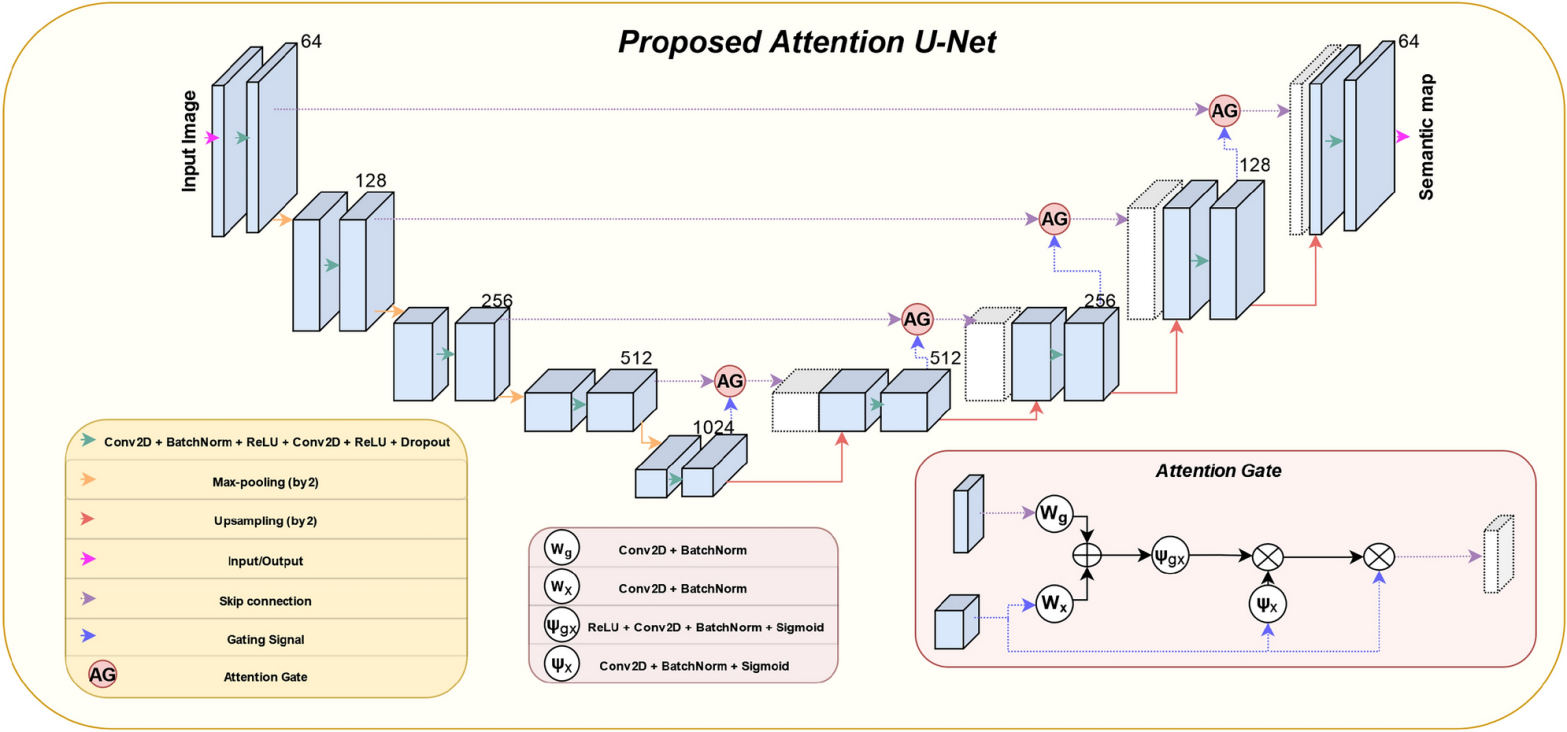
The architecture of proposed U-Net model.

Figure 5 depicts the proposed Attention U-Net architecture. The U-Net architecture^8–11^ is a convolutional neural network (CNN) designed for semantic segmentation tasks. It consists of a contracting path to capture context and a symmetric expanding path for precise localization. The architecture features skip connections between the contracting and expanding paths, enabling the model to preserve spatial information while effectively capturing both global and local features. This design makes U-Net well-suited for tasks such as image segmentation, where pixel-level accuracy and contextual understanding are crucial. The employed architecture incorporates soft attention^9^ on the skip connections, effectively diminishing activations in less significant regions, thus reducing the transmission of redundant features.

The proposed model shows some novelties compared to the state of the art Attention U-Net model presented in^9^, since it uses a deeper architecture, incorporates dropout regularization, and a slightly modified attention gate.

As presented in^12,13^, applying dropout regularization in convolutional networks can improve the model’s accuracy, especially when the training dataset is relatively small. Furthermore, dropout regularization can significantly enhance the generalizability of the model.

In attention gates (AG), a transformation $\Psi _X$ is applied, which has been found to improve performance based on empirical results. The output of AGs is the element-wise multiplication of input feature maps ($x^{l}$), attention coefficients ($\alpha ^{l}$) and input feature modulation term ($\beta _x^{l}$) as follows:

1 $$\begin{aligned} \hat{x}_{i,c}^{l} = x_{i,c}^{l} \cdot \alpha _{i}^{l} \cdot \beta _i^l. \end{aligned}$$

Here, $i$ and $c$ denote the spatial and channel dimensions, respectively. A single scalar attention value is computed for each pixel vector $x_i^{l}$ in $F_l$, where $F_l$ corresponds to the number of feature maps in layer $l$. A gating vector $g_i$ is used for each pixel $i$ to determine focus regions. The additive attention mechanism^14^ is formulated as follows:

2 $$\begin{aligned} q_{att}^{l}&= \psi _{gx}^T \left( \sigma _1 \left( W_x^T x_i^l + W_g^T g_i + b_g \right) \right) + b_{\psi _{gx}}, \end{aligned}$$

3 $$\begin{aligned} \alpha _i^l&= \sigma _2 \left( q_{att}^{l} (x_i^l, g_i; \Theta _{att}) \right) , \end{aligned}$$

4 $$\begin{aligned} \beta _i^l&= \sigma _2 (\psi _{x}^T x_i^l + b_{\psi _{x}}), \end{aligned}$$

where $\sigma _1(x_{i,c})$ corresponds to the rectified linear unit activation function and $\sigma _2(x_{i,c})$ corresponds to the sigmoid activation function. AG is characterized by the parameters of $\alpha _{i}^{l}$, denoted as $\Theta _{att}$, and the parameters of $\beta _{i}^{l}$. The $\Theta _{\text {att}}$ contains linear transformations $W_x$, $W_g$, $\psi _{gx}$, and bias terms $b_{\psi _{gx}}$, $b_{g}$, while $\beta _{i}^{l}$ is characterized by the parameters of linear transformation $\psi _{x}$ and bias term $b_{\psi _x}$. The transformations of the AG gate act on an input feature map $X$, allowing certain parts of the input to receive more emphasis. The outputs of $\Psi _X$ and $\Psi _{gx}$ transformations, are then combined, enabling the model to dynamically adjust its focus based on both spatial and contextual information. This specific attention mechanism enhances generalization by dynamically adjusting feature importance using $\alpha _{i}^{l}$ and $\beta _i^l$. The $\beta _i^l$-transformed input feature map further refines feature selection by modulating the relevance of input features, effectively suppressing less informative regions. By incorporating this additional gating step, the model mitigates overfitting, enhances robustness, and improves generalization, leading to better performance on unseen data.

### Loss function

During training, a weighted loss function is utilized, combining the Cross Entropy^15^ (CE) and the Generalized Dice Loss^16^ (GDL) with weights of 0.3 and 0.7 respectively. By employing these loss functions, specific weight values can be assigned to individual classes, thereby placing greater emphasis on certain classes, such as the weld class. The used weights are 0.1, 0.25, 0.3, and 0.35 corresponding to the background, gap, pin, and weld classes.

The GDL calculates the dissimilarity between predicted and ground truth segmentation masks in semantic segmentation tasks, and it is computed as follows:

5 $$\begin{aligned} L_{GDL} = 1 - \frac{2 \sum _{c=1}^{C} w_c \sum _{i=1}^{N} p_{ic} \cdot t_{ic}}{\sum _{c=1}^{C} w_c \left( \sum _{i=1}^{N} p_{ic} + \sum _{i=1}^{N} t_{ic} \right) } , \end{aligned}$$

where $N$ denotes the total number of pixels, $C$ represents the number of classes, $p_{ic}$ indicates the predicted probability of pixel $i$ belonging to class $c$, $t_{ic}$ serves as the binary indicator (0 or 1) for whether pixel $i$ in the ground truth belongs to class $c$, and $w_c$ signifies the weight assigned to each class, computed as the inverse of the square of the proportion of pixels belonging to that class.

The formula for the weighted cross-entropy loss is:

6 $$\begin{aligned} L_{CE} = -\frac{1}{N} \sum _{i=1}^{N} \sum _{c=1}^{C} w_c \cdot y_{ic} \cdot \log \left( \frac{e^{\hat{y}_{ic}}}{\sum _{k=1}^{C} e^{\hat{y}_{ik}}} \right) , \end{aligned}$$

where $N$ refers to the total number of samples in the dataset, while $C$ represents the number of classes in the classification task. The prediction for class $c$ of the $i$-th sample is denoted by $\hat{y}_{ic}$, and the true class label for the $i$-th sample is denoted by $y_i$. Additionally, $w_c$ represents the weight assigned to class $c$.

Finally, the final loss function utilized in the presented experiment is the weighted combination of these two loss functions, that can be described as follows:

7 $$\begin{aligned} L_{weighted} = 0.3 \times L_{CE} + 0.7 \times L_{GDL}, \end{aligned}$$

where the associated weight values are hyperparameters of the algorithm.

### Comparison of different U-Net variants

**Table 1:** Comparison of different U-Net variants. Bold values indicate the best performance for each metric.

Model	Test dataset				Validation dataset		Parameters
	Acc.	Prec.	Rec.	F1	Acc.	Loss	(M)
Vanilla U-Net^8^	0.8656	0.8853	0.8067	0.8365	0.9203	0.2399	**31.03**
Attention U-Net^9^	0.9175	0.9162	0.8977	0.9060	0.9485	0.2304	31.39
**Proposed model**	**0.9462**	**0.9461**	**0.9326**	**0.9391**	**0.9586**	**0.1757**	31.39

**Fig. 6:**
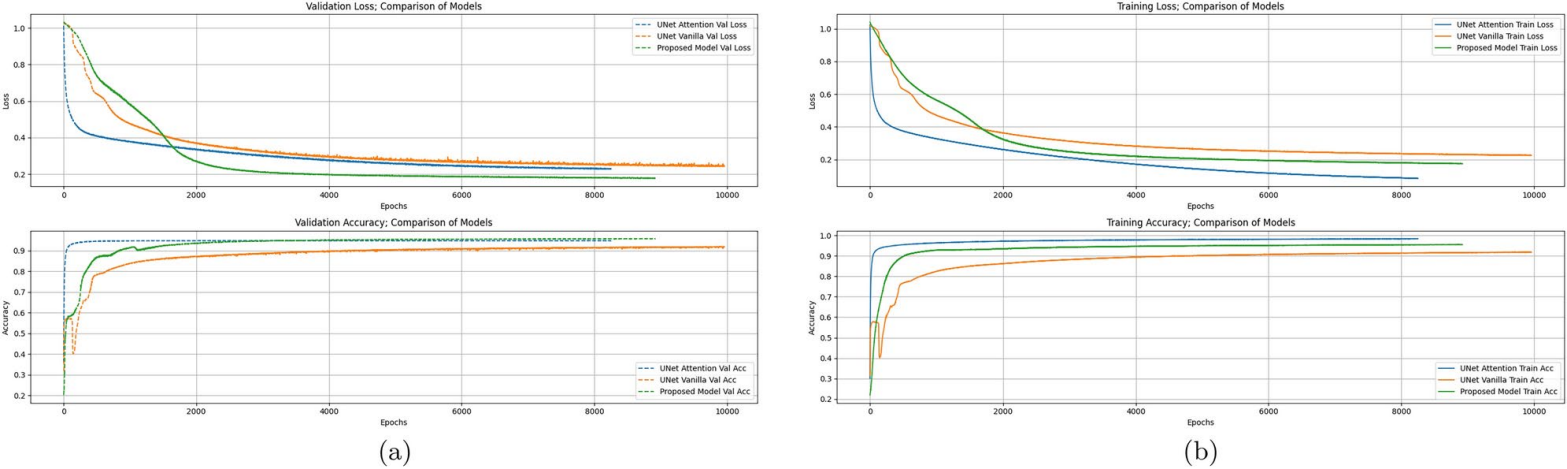
Comparison of training and validation curves for the three models.

Table 1, and Fig. 6 presents the comparison of the Vanilla U-Net model as introduced in^8^, the Attention U-Net model from^9^, and the Proposed Model shown in Fig. 5. The hyperparameter values used for training all three models are as follows: maximum epochs set to 10000, early stopping patience set to 300 epochs, optimizer chosen as Adam, learning rate set to $1 \times 10^{-7}$, weight decay set to $5 \times 10^{-10}$, and batch size set to 50. The models were trained on a system equipped with an Intel(R) Xeon(R) Gold 6248R @ 3.00GHz CPU, two NVIDIA A100-PCIE-40GB GPUs, and 256 GB of RAM.

The learning curves of the models are illustrated in Fig. 6, showing the loss and accuracy values on both the training and validation datasets. Figure 6b presents the training loss and accuracy of the three models throughout the training process, indicating that the Attention U-Net achieves the best performance on the training dataset. However, as shown in Fig. 6a, the Proposed Model outperforms the other two models on the validation dataset. These results suggest that the Proposed Model is more generalizable, benefiting from dropout regularization and the specific attention gate mechanism, while the Attention U-Net shows a tendency to overfit the training dataset.

As the Table 1 and Fig. 6 presents, during the training the Vanilla U-Net model got a 0.9203 validation accuracy and 0.2399 validation loss. The Attention U-Net model got a 0.9485 validation accuracy and 0.2304 validation loss. The proposed model got a 0.9586 validation accuracy and 0.1757 validation loss.

As shown in the Table 1 comparison, the Vanilla U-Net model demonstrates the lowest performance on the test dataset, whereas the Proposed Model surpasses both the Vanilla and Attention U-Net models. The Proposed Model achieves an accuracy of 0.9462, a precision of 0.9461, a recall of 0.9326, and an F1 score of 0.9391. In terms of model complexity, the Vanilla U-Net has the smallest number of training parameters, 31.03 million, while both the Attention U-Net and the Proposed Model have 31.39 million parameters.

## Applied metrics

**Fig. 7:**
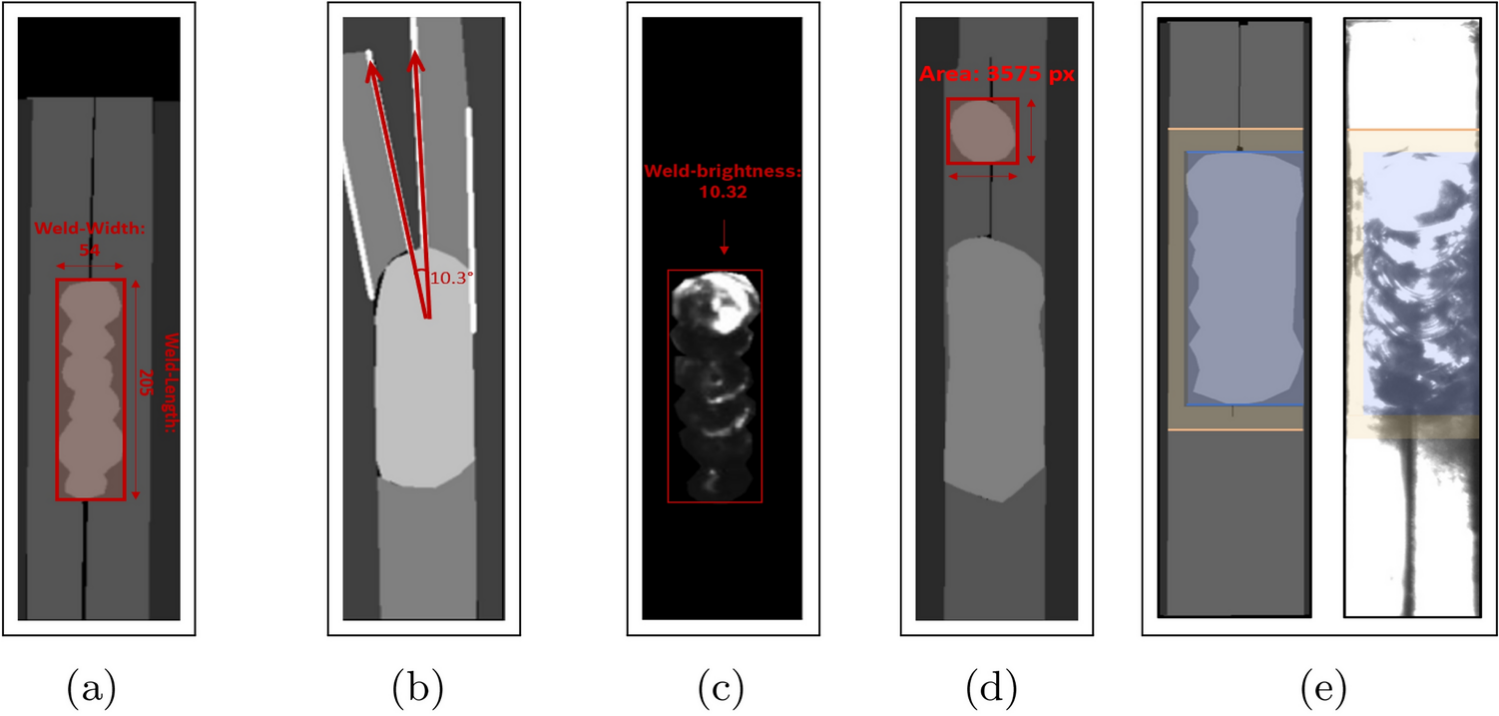
Applied metrics.

The U-Net model, once trained, has the capability to generate a mask for a given input image, assigning each pixel a specific class. To assess the quality of a weld in the input image, various criteria must be satisfied by the corresponding mask. These criteria encompass metrics like the length and width of the weld, its brightness, the contrast between the pin and the weld, as well as the angle of the pin-legs. These metrics are proposed by domain experts, and their proposal is based on the effects or failure modes observed in use cases. For a weld to be accepted, all its metric values must fall within the specified intervals. The interval threshold values are specified and maintained by domain experts.

Specifying intervals isn’t straightforward. The specified boundary values are determined by the model’s accuracy and a cautious approach to ensure that only acceptable items are passed through. Therefore, the challenge is to find appropriate metrics and set a threshold that allows the majority of acceptable items to pass while prevent passing any undesired ones.

The metrics that the presented method implements are the following: weld length, weld width, weld brightness, weld-pin contrast, pin-leg angle and minor-weld area.

- **The length and width values of the weld** (see Fig. 7a) are defined in pixel units. This metric helps filter out cases, for instance, where the weld is not thick enough or the weld length is not long enough, thus not covering the pin perfectly. It’s important to note that these values can be converted to millimeters once the camera calibration parameters are known, a task typically performed by domain experts.
- **Pin angle** (see Fig. 7b) pertains to the angle between the pin legs. This metric aids in identifying instances with bent pin legs. The unit of measurement in this case is degrees.
- **Weld brightness** (see Fig. 7c) assesses the brightness and visibility of the weld within the image. The metric is evaluated as follows: using the predicted mask, the weld is masked out in the original image. Pixel brightness values range from 0 to 255, with black being represented by 0 and white by 255. This metric pertains to the average pixel intensity value of the masked weld within the original image.
- **Minor-weld area** (see Fig. 7d) metric helps in identifying cases where, in addition to the main weld, there exists a smaller weld on the pin. This metric quantifies the area of this minor weld by multiplying its length by its width.
- **Weld-pin contrast** (see Fig. 7e) metric evaluates the visibility of the weld, considering the presence of the pin. Assessing this metric involves utilizing the predicted mask to identify the pixels corresponding to the weld and pin in the original image. Subsequently, the average brightness values are calculated for both the weld and a small neighborhood surrounding the pin. The contrast value is then determined by subtracting the average brightness of the pin from that of the weld.

**Fig. 8:**
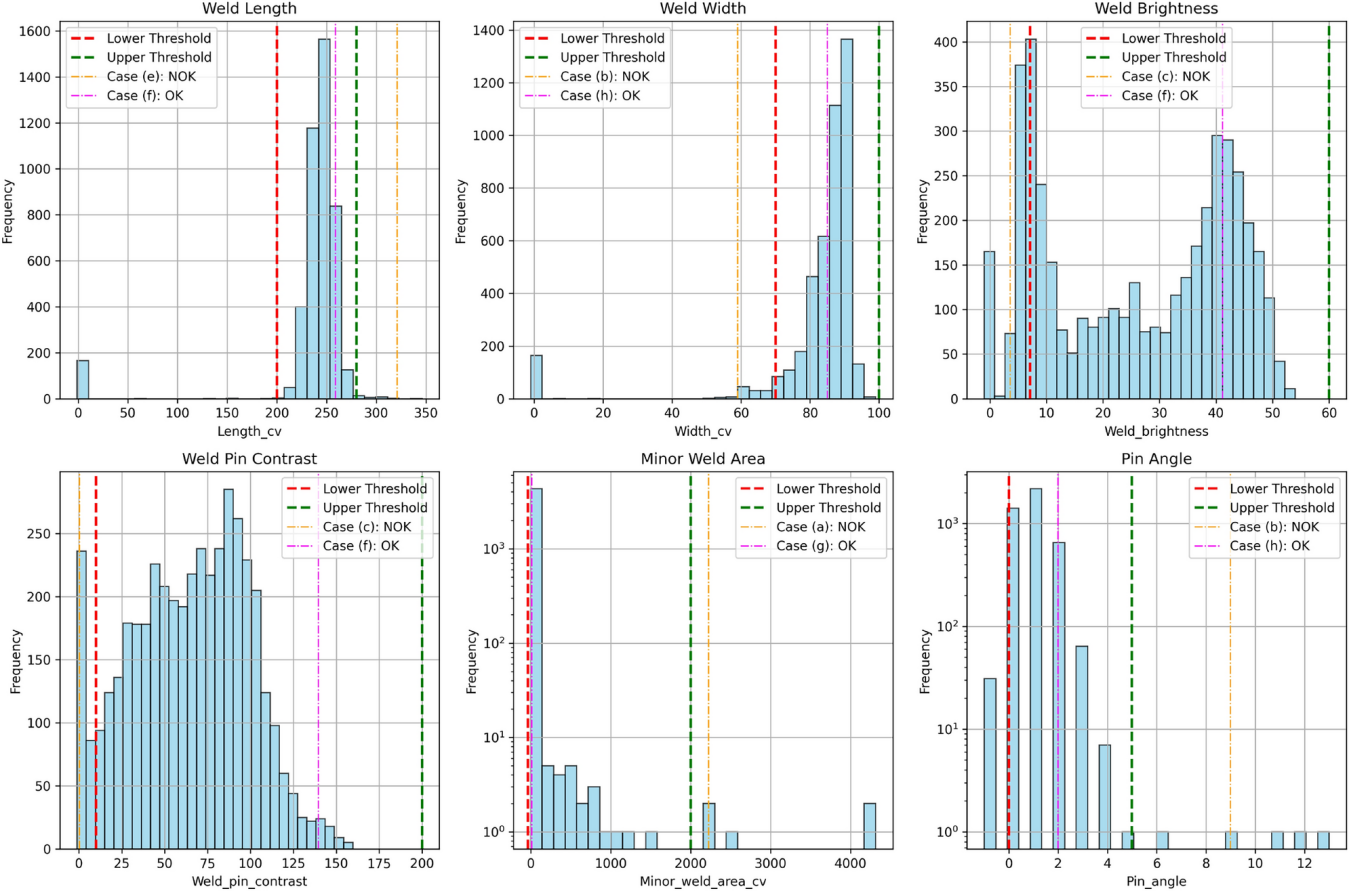
The histogram of each metric.

**Fig. 9:**
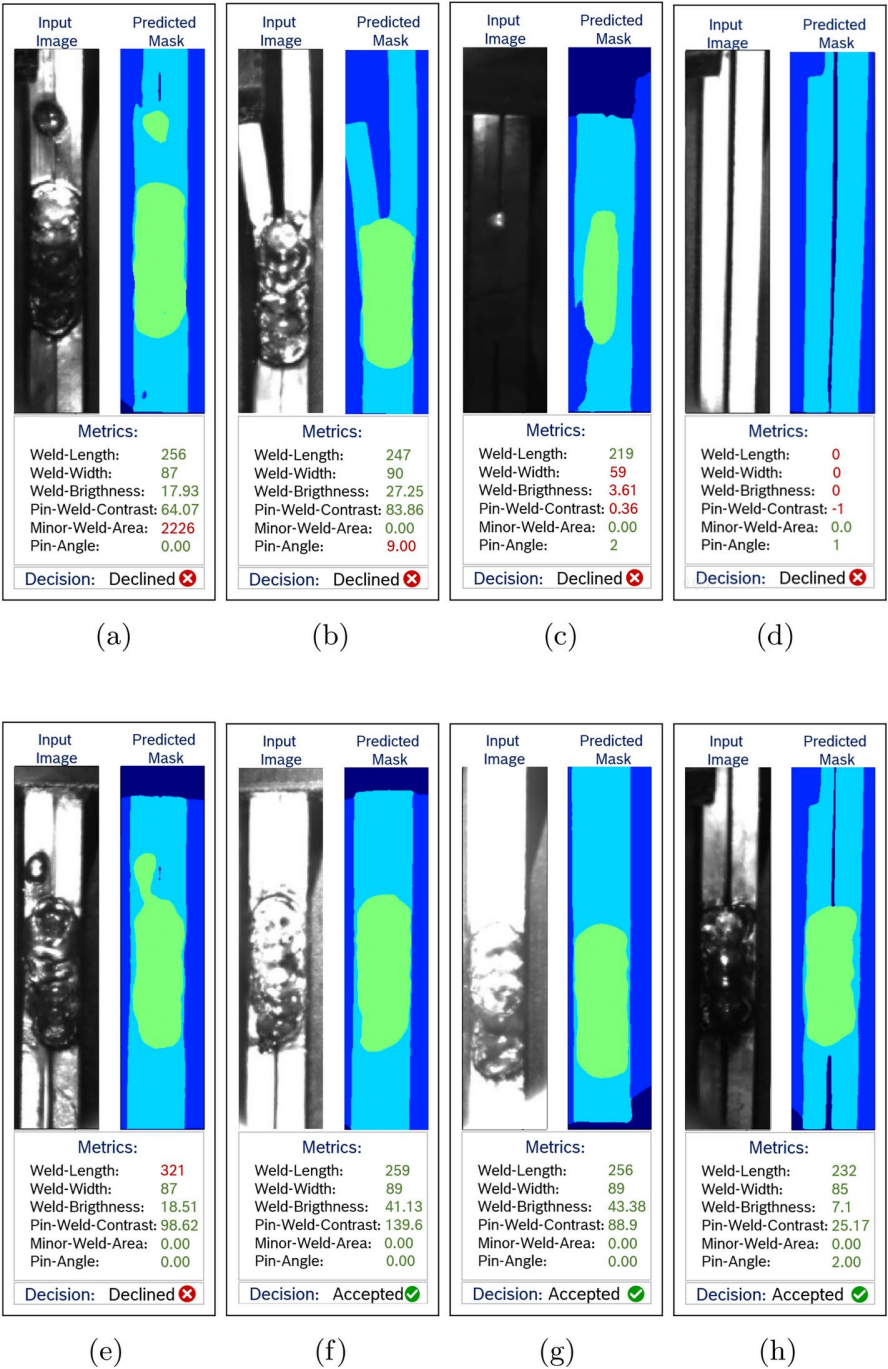
Results of the proposed method.

Figure 8 illustrates histograms for each metric, showcasing the utilized threshold values for the acceptance interval. It also presents several examples for each metric: a value that lies within the acceptance interval and another which lies outside. The cases refer to examples presented in Fig. 9.

In our case, the acceptable range for the length of the weld spans from 200 to 280 pixels, indicating that a weld’s length is considered acceptable if it falls within this interval. Similarly, for the width of the weld, the acceptable range spans from 60 to 100 pixel units. The acceptable range for the weld brightness metric extends from 7.1 to 60, while for the pin-weld contrast metric, it ranges from 7.5 to 200. Additionally, for the pin angle metric, the acceptable range is from 0 to 4.99 degrees. Finally, for the minor-weld area metric, the acceptable range is from 0 to 2000 pixels.

Establishing the threshold values for each metric falls under the responsibility of domain experts, who determine these values through a comprehensive examination of the input image and its corresponding metrics.

In the unseen dataset, 20% of the images were filtered, indicating that approximately 80% were reviewed and approved by the proposed method, while the remaining 20% were forwarded for further investigation by domain experts. This 20% reflects results from our dataset, which was specifically gathered to include more problematic images for training and evaluation purposes. In real-world applications, the need for manual review is much lower. The proposed method has been validated on a dataset of 50,000 images, coming directly from production lines, automating the review process for over 90% of these images. In reality, the proportion of problematic images is approximately 5%. However, the thresholds are set conservatively to ensure that no problematic weldings are mistakenly approved, which accounts for the remaining 10% that may require further inspection. It is important to note that a product may be filtered out based on the image (for example, if it appears too dark), even if the welding itself is correct. This aspect cannot be accurately assessed solely from the image, and a manual inspection by a domain expert may be necessary. After this manual inspection, less than 1% of the actual weldings are filtered out as problematic.

This image-based automation significantly reduces the workload for domain experts, allowing them to focus only on the most problematic cases, and enhances the overall efficiency and productivity in the image review process.

## Results

With the trained Proposed Attention U-Net model for semantic segmentation and the application of the proposed metrics, the automated welding line detection method is now ready to make decisions on the input images. Figure 9 showcases various instances filtered by the provided method. It presents the input images alongside their corresponding predicted masks, associated metrics, and the decision.

In Fig. 9a, a scenario is depicted where a second separate weld is observed on the pin. In such instances, the minor weld area metric will yield a high value, leading to the rejection of the case. In Fig. 9b, an input is depicted where the weld is correct, but the pin leg is bent. The high pin-angle value reflects this fact, thus such cases will be declined. Figure 9c depicts a scenario where the pin and weld appear excessively dark. The model encounters challenges in accurately predicting the mask in such instances, leading to less precise results. These cases will be filtered out for further examination by domain experts. Figure 9d displays an input image with a missing weld. In such instances, the decision will be ’Declined’ due to the values of the length, width, and brightness of the weld, as well as the pin-weld contrast, falling outside the specified intervals.

Figure 9e illustrates a case where the weld length is excessively long, leading to a decline in decision. Figure 9f–h depict cases that are accepted by the proposed method, as all metric values fall within the specified acceptance intervals.

## Workflow

**Fig. 10:**
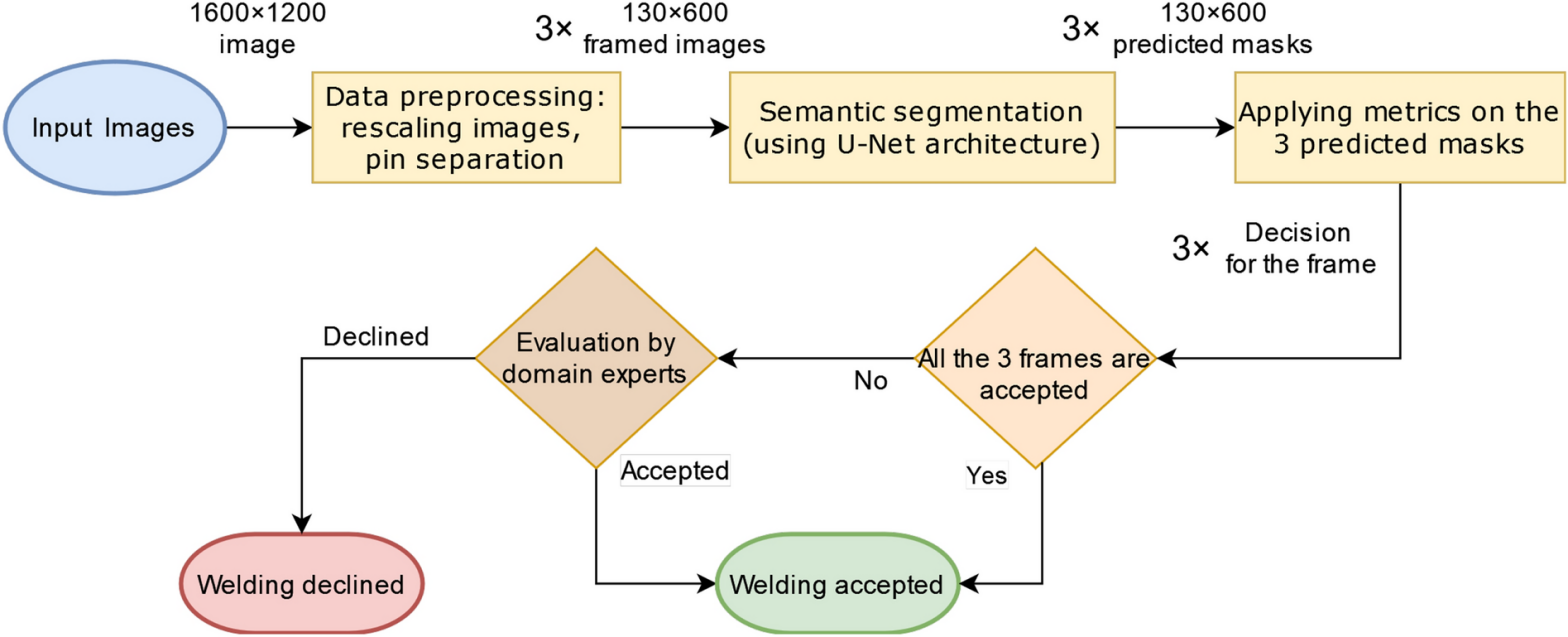
Workflow diagram.

The workflow diagram is depicted in Fig. 10. Initially, the high-resolution input image (refer to Fig. 1) undergoes a data preprocessing step. Consequently, the original image is down scaled and separated into three frames, each focusing on one of the three pins (as shown in Fig. 3). These frames serve as inputs for the U-Net model, which generates a corresponding mask for each frame. Subsequently, metrics are applied to these masks to determine if they meet the specified requirements. Following this evaluation, a decision is made: if all three frames are accepted, the given welding is accepted; otherwise, the decision is passed on to domain experts.

## Conclusion

The method proposed in the article presents a solution to the challenge of identifying welding lines in images, thereby aiding process experts in ensuring product quality and safety. The uniqueness of the proposed method lies in its utilization of a novel Attention U-Net architecture-based model for semantic segmentation, coupled with the integration of rule-based metrics. This approach enables the method to efficiently filter out questionable cases, thereby decreasing the need for manual inspection by more than 90%, enhancing the overall process reliability.

Another advantage of the method is that it enables the implementation of automated AI-based verification by systematically segregating difficult-to-recognize images from the dataset. These are images where both AI and the human eye struggle to discern the content of grayscale images.

The proposed method only considers the data within the three frames. Therefore, if an anomaly occurs between these frames, the current method cannot filter out such data. This limitation highlights a potential avenue for future research. As future work, combining the current solution with a diffusion model could be considered. This diffusion model would be capable of recognizing foreign material and anomalies between the pins.

## Data Availability

The datasets generated during and/or analysed during the current study are available from the corresponding author on reasonable request.
